# Do commencing nursing and paramedicine students differ in interprofessional learning and practice attitudes: evaluating course, socio-demographic and individual personality effects

**DOI:** 10.1186/s12909-016-0605-5

**Published:** 2016-03-03

**Authors:** Karen T. Hallam, Karen Livesay, Romana Morda, Jenny Sharples, Andi Jones, Maximilian de Courten

**Affiliations:** Centre for Chronic Disease, College of Health and Biomedicine, Victoria University, PO Box 14428, Melbourne, Victoria 8001 Australia; Interprofessional Education and Practice Program, Victoria University, PO Box 14428, Melbourne, Victoria 8001 Australia; Discipline of Nursing, College of Health and Biomedicine, Victoria University, PO Box 14428, Melbourne, Victoria 8001 Australia; Discipline of Psychology, College of Arts, Victoria University, PO Box 14428, Melbourne, Victoria 8001 Australia; Victoria University Interprofessional Clinic, Victoria University, PO Box 14428, Melbourne, Victoria 8001 Australia

**Keywords:** Interprofessional education, Health education, Interprofessional practice, Attitudes, Nursing, Paramedicine, Personality, Socio-demographic

## Abstract

**Background:**

Interprofessional education (IPE) requires health students to learn with, from and about each other in order to develop a modern workforce with client-centred care at its core. Despite the client centred focus of IPE, training programs often utilize standard approaches across student cohorts without consideration of discipline, sociodemographic and personality variability that attract students to different health disciplines. Knowing the students who engage in IPE to tailor training may prove as beneficial as knowing the client to delivered individualized client centred care in interprofessional practice (IPP). This research investigates whether students commencing undergraduate nursing and paramedicine degrees ener training with existing demographic and personality differences and, if these are associated with different attitudes towards health care teams and interprofessional education.

**Method:**

This online study recruited 160 nursing and 50 paramedicine students in their first week of their undergraduate course. Students completed questionnaires regarding their background, personality (General Perceived Self Esteem Scale, International Mini Markers) and the attitudes towards health care teams scale (ATHCTS) and interprofessional education perception scale (IEPS).

**Results:**

Results show that commencing nursing and paramedicine students are demographically different on education, gender, speaking a language other than English at home (LOTE) and their own experience with healthcare. The results further demonstrate that LOTE, discipline being studied and personality factors play a role in perceptions regarding interprofessional training whilst discipline being studied impacted on attitudes towards health care teams in the workforce.

**Conclusion:**

These results highlight a number of existing personal and psychological differences between individuals who choose to train in these selected professions. This suggests a need for tertiary education IPE programs to move towards tailoring their education to value this student diversity in the same client centred manner that students are asked to develop clinically.

## Background

In 2010, the World Health Organisation [1] identified a global need to consider a shift in health care models and health education pedagogy. The increase in complex and chronic health conditions in developed countries and a chronic undersupply of health workforces in developing regions has necessitated the re-consideration of the biomedical health model. This new approach requires a broader social context to be considered in health care provision including the biological, medical, social, psychological and community context. Interprofessional practice (IPP) holds many advantages as an emerging health model. Interprofessional practice differs from multidisciplinary approaches in the focus on the interdependence of health care professionals in providing clinical care over and above effective collaboration. It is argued that this approach leads to improved health care outcomes for patients, cost savings in health care provision, reduced negative events and greater health care worker satisfaction [2].

To meet the training needs for developing an interprofessional workforce the tertiary education system globally is moving towards Interprofessional Education (IPE). Interprofessional education is a pedagogical shift in health care education that teaches students to work collaboratively with other health workers in a team environment. The Centre for Advancement in Interprofessional Education (CAIPE) [3] operationalize this as ‘students from at least two different disciplines learning with, from and about each other to improve collaboration and quality of care’. Research is now producing evidence on a range of changes associated with interprofessional education in relation to attitudes and beliefs around IPP. In a recent analysis, Thistlethwaite and colleagues [4] argue for a realist approach to IPE that further elucidates what works and for whom rather than just identifying overall changes in attitudes or simple outcomes.

Sergeant [5] argued for a new way of thinking about IPE. In essence, Sergeant identified social psychology (the study of social interactions and groups) as an important contextual factor. This fits well within Thistlethwaite’s model [4]. In essence, it is argued that theories in social psychology relating to how individuals see themselves, teams and their environments require integration into understanding the outcomes and approaches to IPE. The aim of this research is to incorporate an exploration of just two of these important contextual factors into assessment of interprofessional attitudes and beliefs to develop more comprehensive assessment approaches as called for by field leaders [4, 6]. These psychological factors include assessing the impact of social identity and individual differences on attitudes and beliefs towards interprofessional education in commencing students.

Researchers [7–9] have argued that the Social Identity Approach [10–12] provides a useful framework for understanding interprofessional group processes within a health context. The Social Identity Approach comprises social identity theory (SIT) and self-categorisation theory (SCT) [8, 10–12]). Tajfel [10] identified social identity as the part of a persons self-concept that is derived from their role in social groups and the importance they place on this membership. Individuals belong to multiple groups and thus their identity is fluid and the salience of different aspects of their identity is said to be dependent on contextual factors [7, 11]. A key tenet of the Social Identity Approach is that individuals evaluate more favourably groups of which they are a member and tend to evaluate other groups less favourably [8, 11]. This in-group categorisation and bias can lead to group competition and conflict [7, 8, 11]. Intergroup conflict can also result from perceived differences in power and status that reflect broader contextual influences [11]. It may be important to acknowledge these processes when designing and implementing IPE.

An individual’s identification with their profession is said to be an important component of social identity [8]. Each profession has its own cultural frame of reference shaped by core values, norms, education, training and socialisation [8, 13, 14]. Socialisation into a profession begins very early on in a student’s life and researchers have argued that even first year health care students have relatively strong professional identities and favour their own profession over others [14–16]. Indeed, Michalec et al. [9] found in their investigation of health care students’ attitudes towards their own and other professions that there was significant in-group favouritism. Thus two major challenges in implementing successful IPE programs is to acknowledge and overcome professional in-group biases [7, 8, 17].

It has been argued the development of superordinate healthcare team goals and identity could be an effective means of overcoming professional in-group favouritism [7–9, 14]. Development of a collaborative team identity goes beyond providing opportunities for working with other professional groups as part of IPE training. Individuals must develop interprofessional cultural competence and begin to perceive themselves as part of a superordinate health care team that includes diverse professional groups [14, 17]. This involves a “flexible (re) construction of identity” [8] in that professional subgroups are valued but these subgroups feel that they belong to a team that is working towards common goals. The advantage of the interprofessional education approach is that this construction of health care team identity can be developed through pedagogical approaches and training.

In a comprehensive review by Oandasan and Scott [18] the authors establish the ideal pedagogical framework for effective interprofessional education of student learners. Issues such as educational theory, learning environments, experiential learning and other teaching approaches provide an effective framework for learning interprofessional practice. Others highlighted the important role of factors such as gender and personality in quality of effort, critical thinking and overall performance levels amongst University students [19]. In particular, openness to experience and extraversion were associated with greater academic outcomes. Similarly, in the medical context medical practitioners showing greater levels of conscientiousness and extraversion on the five-factor model were less prone to occupational burnout, dissatisfaction and daily stress [20]. In contrast, those high on indicators of neuroticism were more disposed to these states and also engage less comprehensively with their learning. Similar contrasts were discovered within commencing nurse student groups where different personality types and relationship with feminine/masculine roles were predictive of approach to education that nursing students responded to. Overall, these studies support the proposal by Thistlethwaite and colleagues [4] contending that we must look more completely at the contextual factors surrounding interprofessional education outcome research rather than ascribing changes to the intervention *per se*.

The current research investigates two distinct populations of commencing undergraduate students, namely those in nursing and paramedicine. Research highlights potential differences between these groups. One study revealed nursing students with higher extraversion levels were less likely to succeed in their education [21]. In contrast, others demonstrated that male (but not female) paramedics ‘on the job’ with higher extraversion ratings and lower neuroticism scores were better able to engage in good decision-making [22]. This indicates potential underlying personality style differences between the professions and potentially differences in learning needs.

The aim of this investigation is to determine potential differences between nursing and paramedicine students in terms of background, personality factors and group identification measures and how these impact perception of interprofessional education and attitudes towards interprofessional health care teams. It was hypothesized that there may be significant demographic differences between individuals who have just enrolled in a nursing degree versus those who have enrolled in a paramedicine degree. The research hypothesized that personality differences may occur between these groups of students. The study also investigated the impact of demographic and personality characteristics on student’s attitudes towards interprofessional education and attitudes towards interprofessional health care teams.

## Method

### Participants

This study recruited a total of 210 students enrolled in either nursing (*n* = 160 of an eligible 470 students) or paramedicine (*n* = 50 of an eligible 238 students) courses. The students were in their first week of either a paramedicine or nursing undergraduate course (before any exposure to IPE) at Victoria University.

### Instruments

#### Demographics

Demographics were assessed for all respondents. These included variables that have a known impact on interprofessional attitudes and approach. These included gender, age, education, health discipline, previous experience (working or volunteering) in a health field, having an immediate family member working/volunteering in a health field and using a language other than English (LOTE) at home.

#### General Perceived Self Efficacy Scale (GPSES)

The GPSES was originally developed in the German population and has since been developed into a measurement tool for 33 languages [23]. This scale has become widely used in the assessment of self-efficacy due to the 10 items providing a unidimensional measure of the concept. Psychometrically, the scale has one item that all ten items load toward with strong internal consistency [24] and test-retest reliability. An individual may rate between 10 and 40 on this scale with higher scores indicating greater self-efficacy.

#### International big 5 mini markers test

The five factor model of personality is internationally the most widely used measure of personality. These five factors include conscientiousness, extraversion, intellectual focus/openness to experience, emotional stability and agreeableness which are all rated with eight items to yield individual scores between 8 and 40 (with higher scores being more like the factor labeled, e.g. extroverted). The international big five mini markers test [25] was specifically selected as it was tested on an international population who all studied in English language classes, as do our student population. This scale, albeit brief at 40 items, demonstrates excellent psychometric properties.

#### Attitudes Towards Health Care Teams Scale (ATHCTS)

The ATHCTS [26] was modified from the previous longer scale [27]. This 14-item scale provides a measure of two factors according to a principle component analysis; quality of care (11 items) and cost of team care (3 items). This two-factor structure displays good internal consistency with chronbach alpha levels of 0.83 across four health science disciplines. The response range for the factors is between 11 and 55 for quality of care and 3–15 for cost of team care with higher scores on both scales indicating more positive attitudes.

#### Interdisciplinary education perception scale (IEPS)

The IEPS was originally an 18 items assessment tool with four sub scales [28]. This was later refined to a three sub-scale, 12 item questionnaire [29]. The potential range of an individual’s total score for the IEPS is between 12 and 72 and sub scales score ranges between 5 and 30 (competency and autonomy and perception of actual co-operation scales) and between 2 and 12 on the perceived need for co-operation scale. On all scales, higher scores indicate more positive perceptions. Total scores on this scale were used in addition to the three including Competency and autonomy (ICC 0.58), perceived need for cooperation (ICC 0.6), perception of actual cooperation (ICC 0.57) [29]. These scales provide useful data relating to a number of elements of the Victoria University interprofessional education model, namely working collaboratively, being competent in interprofessional practice, cooperation and autonomy.

### Procedure

Following ethical approval of an online (qualtrics) questionnaire package, the study portal was launched. All students in their first week of their first year of nursing or paramedicine undergraduate degrees were approached before and after lectures and asked to complete the online questionnaire. Further to this, general e-mail reminders and paper advertisements were placed around the three relevant campuses of the University. As students were in first year of their studies and potentially unfamiliar with research, it was highlighted in all contact that participation was completely voluntary and unrelated to their course or class. Students were provided the opportunity to get a brief summary of their own scores on the personality and self-efficacy scales as an acknowledgement for their time. This feedback was compiled by a clinical psychologist and involved providing scores and a key showing which categories this may relate to (ie high, medium or lower self efficacy scores).

### Statistical analysis approach

Completed questionnaire packages were exported from Qualtrics to SPSS version 21. Results were assessed for any violations of the assumptions of parametric analysis. Ten outliers were identified across the seven assessments. Each outlier was only aberrant on one scale so their other test results remained unaltered. These outliers fell more than 1.5 S.D. from the mean and were re-categorized as excluded values. Following the exclusion of the outliers, normality and skewness were re-assed and proved to be in the safe region for all measures.

The initial analysis to assess background differences between groups utilized chi squared (for nominal demographic variables) and univariate ANOVA for continuous demographic variables. The analysis of data related to specific scales was conducted as follows.

### Personality and self-efficacy differences

Personality dimensions on the big five measure are interrelated, hence MANOVA was utilized in statistical analysis to assess the five factors. Bonferroni corrections were utilized to ensure stability of experiment-wise type-1 error rates. Self-efficacy was measured independently using univariate ANOVA.

### Analysis of Interprofessional Education Perception Scale (IEPS) and Attitudes towards Health Care Teams Scale (ATHCTS)

To evaluate the impact of demographic, personality and course variables on the ATHCT and IEPS measures, a multiple regression was performed for each of these measures and subscales. The multiple regression of the IEPS total score included the following selection factors; course enrolled in, gender, language spoken at home, family working in health, volunteer or paid experience in health, general perceived self-efficacy score and each of the big five factors of personality.

### Ethics consent and permissions

The study commenced after receiving full ethical approval by the Victoria University Human Research Ethics Committee (VU-HREC). All participants in this research provided Informed Consent after reading a Participant Information Form outlining the risks and benefits of the research.

## Results

### Response rates

The respective sample sizes of 160 (nursing undergraduate) and 50 (paramedicine undergraduates) were drawn from a total potential participant pool of 470 nursing and 238 paramedicine commencing undergraduates in first year. This represented a response rate of 34 % for nursing and 20 % for paramedicine students and an overall response rate of 29.7 %.

### Demographic differences

The result of the chi squared tests around demographic data are depicted in Table 1.

**Table 1:** Demographic differences between paramedicine and nursing students

Comparison	Comparison	Nursing	Paramedicine	P-value
Gender^a^	Male	19 %	44 %	.001
	Female	81 %	56 %	
Age (S.D.)^b^		23.09 (7.1)	24.4(7.2)	.229
Education^c^	< Year 12	1.3 %	6 %	.017
	Year 12	65.0 %	40 %	
	Incomplete U/G	15.8 %	26 %	
	Undergraduate	15.8 %	16 %	
	Postgraduate	1.9 %	12 %	
History working/volunteering health context ^a^		39 %	56 %	.024
Immediate family member working in health		42 %	50 %	.198
Speaks language other than English at home ^a^		31 %	2 %	<.001

^a^Denotes significant differences on chi square tests between groups at α = .05

^b^Denotes no significant differences between groups on Univariate ANOVA

^c^Denotes significant difference on Univariate ANOVA at α = .05

### Personality differences

**Fig. 1:**
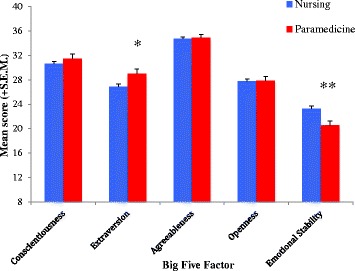
Results on the personality variables for paramedics and nurses. Note. Higher scores on emotional stability scale are associated with increased neuroticism levels. * indicates significant difference at *p* = .05 level. ** indicates significant difference at *p* = .01 level

The omnibus MANOVA results indicated a significant overall difference in personality profile between the nursing and paramedicine students surveyed in this study, *F*(1,206) = 2.822, *p* = .017, eta^2^ = .065, power .830. The applied bonferroni correction leaf to an alpha level of .01. Analysis of the individual factors indicated differences in scores between nursing and paramedicine students in extraversion and emotional instability, with emotional instability being the only factor demonstrating a significant difference between the two experimental groups after Bonferroni correction. This difference is displayed in Fig. 1 alongside the results of nursing and paramedicine students on the other personality variables.

The results of the general perceived self-efficacy scale also showed a significant difference in self efficacy scores between nursing (*n* = 159, M = 31.9, S.D. = 3.7) and paramedicine students (*n* = 50, M = 33.4, S.D. = 3.7), *F*(1,207) = 5.596, *p* = .019, eta^2^ = .026, power = .653.

### Impact of demographic, personality and course selection differences on IEPS and ATHCH measures

#### Interprofessional Education Perception Scale (IEPS) Analysis

The results of the IEPS multiple regression for nursing (*n* = 152) and paramedicine (*n* = 47) students indicated a significant, albeit modest regression equation, *F*(7, 190) = 3.533, *p* = .001, *R*^*2*^ = .115 based on a three factor structure. The significant predictors of this equation were speaking a language other than English at home (*β* = −3.755, *t*(190) = −2.047, *p* = .042), whether students were enrolled in paramedicine or nursing (*β* = 1.296, *t*(190) = 2.072, *p* = .040) and score on the perceived self efficacy scale (*β* = .499, *t*(190) = 2.455, *p* = .015). The results of the first two (dichotomous variables) appear in Table 2.

**Table 2:** Means and S.D. scores of the significant predictors of IEPS scores (dichotomous variables)

Significant variable in model	Response	Mean	S.D.	95 % confidence interval
Language spoken at home	English	60.7	9.7	59.1–62.3
	LOTE	54.8*	11.5	51.4–58.2
Course enrolled in	Nursing	58.1	10.6	56.4–59.8
	Paramedicine	63.2**	8.9	60.7–65.9

*p = .042

**p = .040

In relation to IEPS sub-scales, the results demonstrated modest but significant effects of the variables studied and perceptions around interprofessional education. Specifically, the regressions showed differences in competency and autonomy, *F*(7, 190) = 4.623, *p* < .001, *R*^*2*^ = .146, perceived need for co-operation, *F*(7, 190) = 2.667, *p* = .012, *R*^*2*^ = .089 and perception of actual co-operation, *F*(7, 190) = 3.148, *p* = .004, *R*^*2*^ = .104, sub-scales. Table 3 highlights which of the predictor variables lead to these findings.

**Table 3:** Multiple regressions on IEPS sub-scale score outcomes

	Competency and autonomy sub-scale			Perceived need for co-operation sub-scale			Perception of actual co-operation		
Variable	*B*	*S.E. B*	*β*	*B*	*S.E. B*	*β*	*B*	*S.E. B*	*β*
(Constant)	16.361	4.982		5.545	2.322		13.863	5.063	
Course	.743	.270	.207^a^	.161	.126	.099	.348	.274	.098
Previous health experience	1.239	.646	.134	−.278	.301	−.066	.973	.657	.106
LOTE at home	−1.802	.792	−.167^a^	−.480	.369	−.099	−1.588	.805	−.149^a^
GPSE	.196	.088	.160^a^	.079	.041	.142	.235	.089	.194^a^
Emotional Stability	−.059	.062	−.067	.065	.029	.164^a^	.049	.063	.057
Extraversion	−.029	.058	−.035^a^	.022	.027	.059	.046	.059	.057
R^2^	.146			.089			.104		

^a^denotes a significant factor in affecting relevant sub-scale of the IEPS at α = .05

#### Attitudes Towards Health Care Teams Scale (ATHCT) Analysis

In contrast with the IEPS results, the stepwise analysis of the ATHCT scale indicated a one-factor model. This model indicated that only whether students were enrolled in nursing (*n* = 147) or paramedicine (46) significantly impacted on results for the interprofessional education perception scale results *F*(1, 191) = 4.601, *p* = .033, *R*^*2*^ = .019. An analysis of mean scores indicates that this is associated with a stronger attitude towards health care teams in the paramedical student group (M = 20.7, S.D. = 3.2) than nursing students (M = 19.5, S.D. = 3.4).

## Discussion

The results of the study largely support the importance of considering personal, psychological and discipline factors in interprofessional education. The first hypothesis that there may be demographic differences between nursing and paramedicine students enrolled at Victoria University was supported. The data indicated that nursing students were more likely to be female, slightly younger and markedly more likely to speak a language other than English at home. In contrast, paramedicine students were more likely to have progressed further in other studies and to have volunteered or worked in health before. This result indicates a unique profile pattern may differentiate students beginning different health science courses such as nursing and paramedicine. The high rates of female enrolments in the nursing course at Victoria University is in line with worldwide findings that nursing is significantly more appealing to female than male students [30, 31]. This contrasts markedly with the relatively comparable gender ratios observed in the paramedicine group. The significant number of nursing students who speak a language other than English at home highlighted a potential educational need in the interprofessional curriculum as learning styles vary considerably between cultures [32] and English as a second language students can struggle more academically [33]. In contrast, evidence indicates that living in two cultures may be a strength [34] that can be drawn upon in learning and interactive activities to increase intercultural awareness and improve perspective taking. Despite this concern, the rates of LOTE spoken at home in nursing students are comparable to that observed in the northwestern region of Melbourne (31 % versus 35.8 % derived from Australian Census, 2011).

The second hypothesis relating to personality differences between these cohorts also revealed significant differences between the groups on extraversion and emotional stability (neuroticism) factors. As would be expected from previous research on volunteer paramedics, paramedicine students scored lower on neuroticism measures [22, 35] and higher scores on extraversion [35]. While nursing student scores on neuroticism and introversion were higher than paramedicine students, it is important to note that the overall profile of nursing students is highly balanced with stronger results on agreeableness and openness than other scores for this group, indicating the team oriented nature of both student cohorts despite specific differences. Differences in neuroticism scores between the groups may further represent changes in personality that occur in young adults as a result of life experiences [36]. The demographic highlight variations in cultural diversity, age and gender between the groups that would potentially be associated with different personality profiles.

The finding of higher levels of general perceived self efficacy in paramedicine students is unsurprising when considering that this group also obtained lower scores on the emotional instability scale (i.e. lower neuroticism) and higher scores on extraversion in the big five analysis. These two factors have a clear association with increased sense of self-efficacy in the literature [37]. Fortunately, core skills training in interprofessional education (e.g. communication training) can foster change in individual factors like self efficacy [38]. Interprofessional education programs have a unique opportunity to develop this skill as previous research has demonstrated improvements in work related self-efficacy around communication and client centered care are possible with structured training approaches [39] and interprofessional training leads to greater improvements in self efficacy than traditional training methods [40].

This study investigated the impact of a range of demographic and personality factors on students attitudes towards interprofessional education and attitudes towards interprofessional health care teams. The results indicating that speaking a language other than English at home, undertaking nursing and, to a lesser degree, lower self efficacy led to significantly lower scores on the Interprofessional Education Perception Scale (IEPS) before students commenced IPE training. When analyzing the sub-scale scores on the IEPS the results indicated a modest but significant effect of a range of variables on the competency and autonomy scale (where course enrolled in, LOTE at home, self efficacy and extraversion all positively influenced competency and autonomy scores), perceived need for co-operation (associated with higher neuroticism scores) and perception of actual co-operation (impacted by LOTE at home and self efficacy. Caution must be taken when interpreting such modest associations but it is notable that this result is typical of psychological research on complex phenomena where multiple factors impact an individuals personal beliefs and attitudes. Within this context, being able to account for between 8 and 14 % of variability on the IEPS sub-scales with such a small set of demographic and personality factors illustrates the background variability and inter-individual variability that might impact on approaches to interprofessional education that are currently not accounted for in IPE curriculum.

Notably, attitudes towards health care teams (i.e. more focused on interprofessional practice) reflected only a one-factor model with paramedicine students showing more positive attitudes than nursing students. Some reasons for this finding may include that these students indicate they have had greater exposure to health care personally as a worker or volunteer or exposure in other tertiary training.

There are a number of limitations inherent in this research. The first of these relates to the specificity and sensitivity of the interprofessional measures. Whilst personality measures are generally associated with strong support for their utility over a range of research settings, the interprofessional measures have a briefer developmental history and been criticized for their design (see [40] for review). In addition to this, the research is observational and only assesses perceptions before any interprofessional education. Whilst this provides a baseline measure of where people begin and possible reasons for differences between these groups at baseline, it is only with the continued re-assessment of these students over the coming years and experiences that we will be able to see how these differences impact on their overall outcomes, if at all. In this regard, this research aims to follow students through their learning in terms of assessing perceptions, skills and behavioural change and the impact of these on clients in the clinical context. This study is the necessary first step of a long journey of scientifically researching these students across their training and early careers and should not end with reporting of differences at this stage in student beliefs and attitudes. Finally, practical limitations of this research have limited the research to only those enrolled in nursing and paramedicine studies. We have in fact begun assessment of students in seven health disciplines and data collection will continue for the next four years to create data that provides a better understanding of the personal factors that may attribute to IPE across the health science spectrum.

### Impact of these findings on curriculum design and delivery

Interprofessional capabilities that direct interprofessional learning outcomes focus on teamwork, communication, role boundaries, negotiating interprofessional conflict and reflection on practice [4, 41, 42]. Successful interprofessional curriculum must leverage the discipline specific knowledge of participant students into a theoretical and practical learning agenda that emphasizes the different IPE capabilities. This research highlights that curriculum design should further consider the personal and social attributes of the participant disciplines to develop learning and teaching activities that will foster achievement of the IPE learning outcomes.

Understanding that this learning occurs at the intersection of multiple differences in personal, social and professional identities yet focuses on teamwork, communication and conflict suggests that the content address difference with strategies that support skills to negotiate the pedagogical shift to collaborative work and shared goal setting in client management. In developing this content the curriculum designer must focus on the differences rather than the collective end point.

Cross-cultural communication strategies provide emphasis on curiosity and difference and have applicability to maintaining relationships through negotiation to develop shared understanding [43, 44]. Practical activities need to emphasize the acknowledgement that the gestalt of care is greater than the contribution of each member of the team but differs from interdisciplinary or multidisciplinary care through development of agreed care priorities rather than aligned yet different care objectives. Through exploring team differences in culture, approach and style we aim to develop better treatment plans and supports for clients. Training needs to capture this difference and common goal if IPE is to fulfill its place as heir apparent to existing health team models.

Problem based learning exercises and learning through simulation techniques are often used to support development of IP practice. Scenarios should provide authentic examples of the outcomes that are achieved through IP practice with emphasis on the contributions of the individual disciplines. Activities that assist the individual to identify with the IP team as a new entity may reduce discipline based tensions. Equally attention and time need to be applied to relationship building within IP teams.

## Conclusion

These results highlight the potential barriers and opportunities inherent in developing interprofessional education and practice across disciplines, cultures and professional identities. In this sense, interprofessional education should consider garnering the inherent diversity of students to enrich interprofessional curriculum, rather than deliver generic programs suited for the whole. The undergraduate curriculum provides the foundation for developing empathy, cultural sensitivity, sharing ideas and working collaboratively in IPP. Keeping in mind the varied and contextual needs of different students provides educators with knowledge to develop better training but also model the core IPP skills of being client focused to students in the way we utilize, support and foster them as individuals within a team.

### Availability of supporting data

This study forms a section of a larger overall research program that is being conducted between 2015 and 2018. Due to funding restrictions, databases will not be released until this completion date.
